# Lower Thyroid Function and Higher Plasma Choline: Effect Modification by Metabolic Dysfunction-Associated Steatotic Liver Disease

**DOI:** 10.3390/ijms262110525

**Published:** 2025-10-29

**Authors:** Adrian Post, Margery A. Connelly, Stephan J. L. Bakker, Robin P. F. Dullaart

**Affiliations:** 1 Department of Nephrology, University Medical Center Groningen, University of Groningen, 9700 RB Groningen, The Netherlands; s.j.l.bakker@umcg.nl; 2 Labcorp, Morrisville, NC 27560, USA; connem5@labcorp.com; 3 Department of Endocrinology, University Medical Center Groningen, University of Groningen, 9700 RB Groningen, The Netherlands; dull.fam@12move.nl

**Keywords:** thyroid function, choline, TMAO, general population

## Abstract

Evidence is accumulating that there is a bidirectional relationship between thyroid function and the gut microbiome. We assessed associations of gut microbiome-derived circulating metabolites, choline, trimethylamine N-oxide (TMAO), and betaine with thyroid function status. Among 4771 euthyroid participants of the community-dwelling PREVEND cohort study (thyroid stimulating hormone (TSH), free thyroxine, and free triiodothyronine levels within the reference range; no use of thyroid function altering medication), associations of TSH (higher levels indicating low–normal thyroid function) with choline, TMAO, and betaine (determined by nuclear magnetic resonance spectroscopy) were assessed. Plasma choline varied by TSH category with the highest values observed in the highest TSH quartile (*p* < 0.001). Such a trend was also found for TMAO (*p* = 0.10) but not for betaine (*p* = 0.68). Linear regression analysis showed a positive association of choline with TSH in fully adjusted analysis (std β: 0.04 (95% CI, 0.01; 0.07; *p* = 0.012)). TMAO was associated with TSH in unadjusted analysis (std β: 0.03 (95% CI, 0.01; 0.06; *p* = 0.031)), but not in a fully adjusted model (0.03 (95% CI, −0.01; 0.06; *p* = 0.094)). Betaine was not associated with TSH. The association of choline with TSH was more pronounced in participants with an elevated fatty liver index, a proxy of metabolic dysfunction-associated steatotic liver disease (fully adjusted std β: 0.08; 95% CI, 0.03; 0.13; *p* = 0.003). Given associations of higher plasma choline and TMAO with cardiovascular disease and mortality, low–normal thyroid function could influence cardiometabolic health via effects on gut microbiome-derived circulating metabolites.

## 1. Introduction

Thyroid function is considered to affect many prevalent non-communicable disorders, including cardiovascular and metabolic diseases [1,2]. Even low–normal thyroid function, as inferred from plasma thyroid stimulating hormone (TSH) levels in the upper normal range, may be associated with cardiometabolic risk factors, such as changes in the lipoprotein profile and metabolic dysfunction-associated steatotic liver disease (MASLD) [3,4]. Over the past few years, evidence has accumulated that abnormalities in the intestinal microflora may contribute to metabolic disorders [5]. Interestingly, there is evidence for a bidirectional relationship between thyroid function and the gut microbiome. On the one hand, there are observations from mainly animal models which suggest that intestinal microbiota can affect thyroid autoimmunity and thyroid function [6,7], with some intestinal bacterial taxa showing an adverse effect, while others show a protective effect on thyroid function [7]. On the other hand, transplantation of fecal microbiota from hypothyroid patients to mice causes decreases in thyroxine levels [8]. Rat models of hypo- and hyperthyroidism result in thyroid function-specific changes in the gut microbiome [9], while in humans with Graves’ disease changes in the gut microbiome take place after anti-thyroid drug treatment [10]. These findings underscore a complex, bidirectional interplay between thyroid hormone status and the intestinal microbiome, with potential implications for both diagnosis and therapy.

Despite increasing focus on the interrelationship between the gut microbiome and thyroid function status, the possible role of clinically relevant gut microbiome-derived circulating metabolites remains elusive. Among these, higher circulating levels of choline, derived from dietary sources and intestinal bacterial production, have been shown to confer an increased risk of cardiovascular disease (CVD) and all-cause mortality [11]. Choline is involved in the generation of trimethylamine (TMA, N,N-dimethylmethanamine), which in turn is converted to trimethylamine N-oxide (TMAO, trimethylamine N-oxide (N,N-dimethylmethanamine N-oxide)) by flavin monooxygenase3 in the liver [11,12]. TMAO is adversely associated with several cardiometabolic disorders, and is associated with mortality in the general population, in subjects with MASLD, as well as in kidney transplant and liver transplant recipients [13]. Circulating betaine (N,N,N-trimethylglycine), another gut microbiome-related metabolite, was found to be inversely associated with new onset diabetes [14], but was unrelated to CVD and mortality in a recent meta-analysis [11].

Given the interrelationship between thyroid function and the gut microbiome and in the absence of data with respect to the association of low–normal thyroid function (as inferred from higher TSH) and gut-derived circulating metabolites, we sought to determine associations of TSH with choline, TMAO, and betaine, determined by nuclear magnetic resonance (NMR), among euthyroid participants of the community-dwelling Prevention of Renal and Vascular End Stage Disease (PREVEND) cohort study. Furthermore, in view of the role of the gut microbiome in the pathogenesis of MASLD [4] and our previous finding that plasma TMAO is elevated and is associated with mortality in subjects with MASLD, we also assessed whether such possible associations are more pronounced in the context of an elevated fatty liver index (FLI) as a proxy for co-existing MASLD.

## 2. Results

Participants with the highest TSH levels were older, had slightly lower FT4 levels, and had anti-TPO antibodies more frequently (Table 1). They had a modestly higher BMI and waist circumference, smoked less frequently, and consumed <1 drink of alcohol more frequently. eGFR and urinary albumin excretion were slightly lower with higher TSH levels. Plasma choline was gradually higher with higher TSH (*p* < 0.001), and a similar though non-significant trend was observed for TMAO (*p* = 0.099). Betaine did not vary between the TSH categories.

Linear regression analyses showed a modestly positive association of plasma choline with TSH, both in crude and adjusted analyses (Table 2). Likewise, TMAO was positively associated with TSH in unadjusted and partly adjusted analysis, but the association did not achieve statistical significance in fully adjusted analysis. Betaine was unrelated to TSH.

Notably, the association of choline with TSH was stronger in participants with an elevated fatty liver index (FLI; *n* = 3324) than in participants without an elevated FLI (*n* = 1425) (missing values of FLI in *n* = 22) (P-interaction = 0.071; Table 3). Associations of plasma choline and TMAO did not vary by sex (P-interaction = 0.53 and 0.97, respectively; Table S1), nor by anti-TPO positivity (P-interaction = 0.83 and 0.32, respectively; Table S2).

In a sensitivity analysis excluding participants with the highest 5th percentile of choline, a similar association with TSH was found (Table S3). In a sensitivity analysis with additional adjustment for CRP, the associations remained virtually unchanged (Table S4).

## 3. Discussion

This study demonstrates an association between circulating choline—and to a lesser extent, TMAO—and low–normal thyroid function, reflected by higher TSH levels in euthyroid individuals from the community-based PREVEND cohort. The association of choline with TSH was more pronounced in participants with an elevated FLI, a proxy of MASLD. Given the interplay between the gut microbiome and thyroid function, these findings suggest that thyroid status may influence circulating microbiome-derived metabolites, particularly in the context of MASLD.

The metabolism of phosphatidylcholine by gut bacteria leads to the production of choline, which is then metabolized to either TMA or betaine [11]. Choline is a component of membrane phospholipids, plays a role in one-carbon metabolism, and is required for cholinergic neurotransmission, as well as for lipid and cholesterol metabolism [11,15]. Although essential for many biological functions, higher circulating choline levels have been associated with adverse health outcomes [11]. TMA, the precursor of TMAO, is a product of choline and L-carnitine.

Among other potentially adverse effects, TMAO, being cleared by the kidneys, likely plays a role in the development of atherosclerosis by stimulating platelet reactivity. Trimethylamine N-oxide (TMAO) has been shown to exert pro-inflammatory and vascular effects, which could indirectly influence thyroid function through systemic pro-inflammatory pathways. Experimental studies indicate that TMAO activates NF-κB signaling and may engage ERK1/2-related pathways in endothelial cells [16], promotes NLRP3-inflammasome activation and M1 macrophage polarization [17], and shifts the Th17/Treg balance toward a pro-inflammatory state [18]. Furthermore, TMAO may affect endoplasmic reticulum stress [19]. Clinically, higher TMAO concentrations have been associated with obesity and MASLD, conditions characterized by low-grade inflammation [20], suggesting a possible link between elevated TMAO, systemic inflammation, and altered thyroid function.

Like TMAO, circulating betaine is derived from gut microbiome synthesis and dietary sources [14]. Betaine is the oxidation product of choline and serves as a methyl donor in one-carbon metabolism. Betaine affects phosphatidylcholine synthesis, hepatic fat accumulation steatosis, and triglyceride-rich lipoprotein metabolism [14,21], and may improve oxidative stress, inflammation, and apoptosis [14,22]. Such actions of betaine likely contribute to the potential beneficial effects of betaine on metabolic disorders-related health. Notably, no associations of betaine with TSH were found in any of the current analyses.

The association of higher TSH with choline and TMAO did not vary between sexes nor between participants with and without anti-TPO antibody positivity. Of potential interest, the association of choline with higher TSH was suggestively stronger in participants with an elevated FLI compared to participants without an elevated FLI, with an approximately 3- to 4-fold higher standardized regression coefficient in participants with an elevated FLI [23,24,25]. The role of the gut microbiome in the pathogenesis of MASLD is increasingly recognized [23,24,25]. Gut dysbiosis, disruptions in the intestinal barrier, and systemic inflammation are involved in the complex pathogenesis of hepatic fat accumulation, the key feature of MASLD, and its progression to steatohepatitis, hepatic fibrosis, cirrhosis, and hepatocellular carcinoma [26]. Of further interest, lower FT4 levels are associated with an increased risk of MASLD both longitudinally and cross-sectionally [27], while higher TSH levels are associated with an increased risk of fibrosis [4]. Combined, an interaction of an elevated FLI on the association of TSH with circulating levels of choline, as shown here, thus seems plausible in the context of the role of the gut microbiome and thyroid function status in the pathogenesis of MASLD.

In our study, participants with the highest TSH had slightly lower FT4 but similar FT3, and had anti-TPO antibodies more frequently than expected [28], and were also somewhat older and had a higher BMI and waist circumference. Patients with the highest TSH also had lower eGFR and albuminuria, smoked less frequently, and drank less alcohol [29,30,31]. Consequently, we adjusted for age, sex, anti-TPO antibodies, waist circumference, eGFR, albuminuria, smoking, and alcohol use when assessing the association of choline, TMAO, and betaine with TSH in multivariable linear regression analysis. Of further note, thyroid function status may affect the risk of CVD events [32,33]. A recent meta-analysis demonstrated that lower TSH levels are associated with all-cause and CVD mortality, with a J-shaped association between FT4 with such adverse outcomes [34]. Hence, in view of adverse effects on plasma choline and TMAO on cardiometabolic health [11,12,13], it seems plausible to hypothesize that thyroid function status could in part influence cardiometabolic health via effects on gut microbiome-derived metabolites.

The present study has strengths and limitations. First, the present analysis was carried out in a large, extensively phenotyped community-dwelling cohort. Second, we made use of highly specific, well-validated, and high-throughput NMR-based assays to measure choline, TMAO, and betaine. However, dietary intake data of the PREVEND participants were not available, making it so that we could not adjust for variations in diet intake, including choline. Nonetheless, similar associations of choline with higher TSH were found in a sensitivity analysis excluding participants with the highest choline levels, making bias consequent to high choline intake very unlikely. In addition, only strictly euthyroid PREVEND participants who were mainly of North European descent were included, as carried out previously [28]. Therefore, the currently reported associations may differ in (subclinical) hypo- and hyperthyroid individuals and in subjects of other ethnicities. Finally, we performed a cross-sectional association study, which precludes us from revealing causal relationships. Longitudinal studies are required to assess associations of (changes in) thyroid function status on adverse health outcomes taking gut microbiome-derived metabolites into account.

## 4. Materials and Methods

### 4.1. Study Population

This investigation adhered to the ethical principles outlined in the Declaration of Helsinki and received approval from the Medical Ethics Review Board of the University Medical Center Groningen, The Netherlands (reference ME96/01/022). Written informed consent was obtained from all participants.

The design, objectives, and recruitment strategy of the PREVEND cohort have been described in detail elsewhere. In brief, the PREVEND study comprises a population-based sample of mainly Northern European adults from the city of Groningen in the northern Netherlands. For the present analysis, data from the second examination round (2001–2003) were used, including 6892 participants. Individuals lacking baseline data on TSH, FT4, choline, TMAO, or betaine, or using medications that influence thyroid function, were excluded. Only participants with euthyroid status were retained, defined as serum free triiodothyronine (FT3; 3.1–6.8 pmol/L), free thyroxine (FT4; 12–22 pmol/L), and thyroid stimulating hormone (TSH; 0.27–4.20 mIU/L) all within reference limits. The final analytical sample comprised 4771 individuals. A schematic overview of participant selection is provided in Figure S1. Anthropometric and clinical parameters, including body mass index, blood pressure, smoking and alcohol habits, presence of hypertension, and estimated glomerular filtration rate (eGFR), were assessed as previously reported [35].

The fatty liver index (FLI) was used as proxy for the diagnosis of MASLD [36,37,38]. The fatty liver index (FLI) was calculated from BMI, gamma-glutamyl transferase (GGT), triglycerides, and waist circumference data according to the following equation: [e^(0.953 × loge (triglycerides) + 0.139 × BMI + 0.718 × loge(GGT) + 0.053 × waist circumference − 15.745)]/[1 + e^(0.953 × loge (triglycerides) + 0.139 × BMI + 0.718 × loge (GGT) + 0.053 × waist circumference − 15.745)] × 100. An FLI threshold of 60 is generally considered optimal for identifying MASLD, yielding an overall accuracy of approximately 84%, with a sensitivity of 61% and a specificity of 86% when compared against ultrasonographic diagnosis [36]. Accordingly, a threshold of FLI ≥ 60 was applied as an indicator of MASLD. The FLI is recognized as one of the most extensively validated indices for assessing hepatic steatosis in large-scale population studies [38].

### 4.2. Laboratory Methods

Serum and ethylenediaminetetraacetic acid (EDTA)-anticoagulated plasma samples were aliquoted and stored at −80 °C until analysis. Serum concentrations of TSH, FT4, FT3, and anti-thyroid peroxidase antibodies (anti-TPO) were measured using electrochemiluminescent immunoassays on the Roche Modular E170 Analyzer (Roche Diagnostics, Mannheim, Germany). Anti-TPO titers > 34 kIU/L were classified as positive. Fasting plasma glucose was determined by dry chemistry (Eastman Kodak, Rochester, NY, USA), and high-sensitivity C-reactive protein (CRP) by nephelometry (BNII, Dade Behring; Marburg, Germany) with a detection limit of 0.18 mg/L. Gamma-glutamyl transferase (GGT) was measured using standard automated methods.

### 4.3. Quantification of Microbiome-Related Metabolites

Frozen EDTA plasma samples (−80 °C) were shipped to Labcorp (Morrisville, NC, USA) for analyses. Concentrations of the microbiota-derived metabolites choline, TMAO, and betaine were quantified using the Vantera^®^ Clinical Analyzer (Labcorp, Morrisville, NC, USA), a fully automated 400 MHz proton (^1^H) NMR spectroscopy platform applying deconvolution-based algorithms, as described previously [14,39,40]. For pools with lower and higher concentrations of analyte, the choline assay has intra- and inter-assay coefficients of variation (CV%) ranging from 5.4 to 11.3% and 6.4 to 10.8%, respectively [40]. The TMAO assay has intra- and inter-assay CV% ranging from 4.3 to 10.3% and 9.8 to 14.5%, respectively [39]. The betaine assay has intra- and inter-assay CV% ranging from 1.3 to 4.3% and 2.5 to 5.5%, respectively [14].

### 4.4. Statistical Analyses

All statistical analyses were conducted using R software, version 4.3.0 (R Foundation for Statistical Computing, Vienna, Austria). Data are presented as mean ± standard deviation (SD) for normally distributed variables, median [interquartile range] for skewed variables, and number (percentage) for categorical variables.

Comparisons of numeric and categorical data across (sex-stratified) TSH quartiles were performed by one way analysis of variance (ANOVA), Kruskal–Wallis tests, or multinomial χ^2^ tests. Univariable and multivariable-adjusted relationships were carried out by linear regression analyses with the results presented as standard regression coefficients with 95% confidence intervals (CIs). In linear regression analyses, choline, TMAO, and betaine were log_2_-transformed, and TSH was square root transformed in order to better comply with the assumptions of linear regression, specifically normality of residuals and homoscedasticity. Interaction terms were calculated as the product terms of the variables of interest. A two-sided *p* value < 0.05 was considered to indicate statistical significance except for interaction terms for which a *p*-value < 0.10 was considered to indicate statistical significance. A sensitivity analysis was included in which we performed additional adjustments for CRP.

## 5. Conclusions

The present study demonstrated, to our knowledge for the first time, that the intestinal microbiome-derived metabolites, choline, and to some extent also TMAO, are associated with low–normal thyroid function in euthyroid individuals with a suggestively stronger association in subjects with suspected MASLD. In view of the effects of higher plasma choline and TMAO with cardiovascular disease and mortality, we surmise that low–normal thyroid function could influence cardiometabolic health via the effects on gut microbiome-derived circulating metabolites.

## Figures and Tables

**Table 1:** Baseline clinical and laboratory characteristics including choline, TMAO, and betaine according to sex-stratified quartiles of TSH.

Variable	Overall Population	TSH Quartile 1 (Lowest)	TSH Quartile 2	TSH Quartile 3	TSH Quartile 4 (Highest)	*p*-Value
Number of participants	4771	1193	1192	1193	1193	
TSH, mIU/L	1.71 ± 0.81	0.82 ± 0.20	1.33 ± 0.14	1.85 ± 0.20	2.84 ± 0.53	<0.001
Free T4, pMol/L	15.71 ± 1.87	15.98 ± 1.89	15.83 ± 1.87	15.70 ± 1.85	15.33 ± 1.79	<0.001
Free T3, pMol/L	4.86 ± 0.52	4.85 ± 0.51	4.85 ± 0.50	4.87 ± 0.53	4.87 ± 0.53	0.53
Male sex, *n* (%)	2427 (50.9)	607 (50.9)	606 (50.8)	607 (50.9)	607 (50.9)	0.99
Age, years	52.95 ± 11.87	53.78 ± 12.45	52.27 ± 11.44	52.25 ± 11.88	53.52 ± 11.62	0.001
Waist circumference, cm	91.77 ± 12.86	92.06 ± 12.89	90.95 ± 12.48	91.60 ± 13.09	92.49 ± 12.95	0.025
Body mass index, kg/m^2^	26.54 ± 4.33	26.59 ± 4.36	26.23 ± 4.00	26.56 ± 4.37	26.78 ± 4.57	0.019
Alcohol categories (%)						0.001
Never	1149 (24.3)	335 (28.5)	253 (21.4)	271 (22.9)	290 (24.5)	
Less than 1 drink per day	2325 (49.3)	539 (45.9)	585 (49.5)	602 (50.9)	599 (50.7)	
More than 1 drink per day	1245 (26.4)	300 (25.6)	343 (29.0)	309 (26.1)	293 (24.8)	
Current smoking	1344 (28.6)	415 (35.5)	370 (31.4)	318 (27.0)	241 (20.4)	<0.001
eGFR_creat+cys_, mL/min/1.73m^2^	97.61 ± 16.98	97.53 ± 17.65	98.80 ± 16.23	98.11 ± 16.78	95.99 ± 17.12	0.001
Urinary albumin excretion	8.53 [6.02, 15.40]	8.94 [6.07, 18.29]	8.74 [6.01, 15.48]	8.49 [6.01, 14.54]	8.10 [6.00, 14.10]	0.011
Triglycerides, mmol/L	1.10 [0.80, 1.59]	1.09 [0.79, 1.55]	1.06 [0.77, 1.52]	1.09 [0.79, 1.60]	1.19 [0.85, 1.71]	<0.001
Gamma-glutamyltransferase, U/L	24 [16, 38]	23 [15, 38]	23 [16, 38]	24 [16, 38]	24 [16, 39]	0.88
Fatty liver index	36 [15, 67]	38 [15, 66]	33 [14, 62]	35 [14, 68]	39 [16, 69]	0.008
Lipid lowering drugs, *n* (%)	337 (8.2)	84 (8.1)	79 (7.8)	83 (8.2)	91 (8.7)	0.89
Antihypertensive drugs, *n* (%)	859 (20.9)	228 (22.0)	190 (18.8)	212 (20.9)	229 (22.0)	0.24
Antidiabetic drugs, *n* (%)	153 (3.2)	35 (2.9)	33 (2.8)	34 (2.9)	51 (4.3)	0.12
Diabetes, *n* yes (%)	268 (5.6)	69 (5.8)	54 (4.6)	63 (5.3)	82 (6.9)	0.09
hs-CRP, mg/L	1.32 [0.60, 2.96]	1.40 [0.63, 3.12]	1.25 [0.59, 2.81]	1.26 [0.60, 2.86]	1.36 [0.59, 3.11]	0.33
Positive anti-TPO antibodies, *n* (%)	367 (7.7)	56 (4.7)	53 (4.5)	92 (7.7)	166 (13.9)	<0.001
Choline, µmol/L	7.30 [5.80, 9.20]	7.10 [5.70, 9.20]	7.30 [5.70, 9.10]	7.30 [5.80, 9.10]	7.50 [5.90, 9.30]	0.04
TMAO, µmol/L	3.20 [1.80, 5.70]	3.20 [1.70, 5.70]	3.20 [1.70, 5.50]	3.20 [1.70, 5.90]	3.40 [1.90, 5.90]	0.10
Betaine, µmol/L	36.6 [30.7, 43.7]	36.8 [30.8, 43.9]	36.5 [30.6, 43.4]	36.5 [30.8, 43.2]	36.8 [30.5, 44.2]	0.68

Data are presented as means ± SD for normally distributed continuous variables, as medians (interquartile ranges) for skewed continuous variables, or as numbers (*n*) with % in brackets. Abbreviations: TSH: thyroid stimulating hormone.

**Table 2:** Linear regression analyses of thyroid stimulating hormone (TSH) with choline, TMAO, and betaine (*n* = 4771).

	Choline		TMAO		Betaine	
Model	Std. β (95% CI)	*p*-Value	Std. β (95% CI)	*p*-Value	Std. β (95% CI)	*p*-Value
Model 1	0.03 (0.01; 0.06)	0.049	0.03 (0.01; 0.06)	0.031	−0.03 (−0.06; 0.01)	0.054
Model 2	0.05 (0.02; 0.07)	0.001	0.04 (0.01; 0.06)	0.021	−0.01 (−0.03; 0.02)	0.74
Model 3	0.03 (0.01; 0.07)	0.018	0.02 (0.01; 0.06)	0.12	0.01 (−0.03; 0.03)	0.85
Model 4	0.04 (0.01; 0.07)	0.012	0.03 (−0.01; 0.06)	0.094	0.02 (−0.03; 0.03)	0.82

Model 1: Crude; Model 2: Adjusted for age and sex; Model 3: As model 2, additionally adjusted for waist circumference, eGFR and urinary albumin excretion, alcohol intake, and smoking; Model 4: As model 3, additionally adjusted for positive anti-TPO antibodies.

**Table 3:** Linear regression analyses of thyroid stimulating hormone (TSH) with choline, TMAO, and betaine, stratified based on a fatty liver index below or above 60.

Fatty Liver Index ≥ 60 (*n* = 1425)						
	Choline		TMAO		Betaine	
**Model**	**Std. β (95% CI)**	***p*-Value**	**Std. β (95% CI)**	***p*-Value**	**Std. β (95% CI)**	***p*-Value**
Model 1	0.07 (0.02; 0.12)	0.008	0.04 (−0.01; 0.09)	0.14	−0.04 (−0.09; 0.01)	0.14
Model 2	0.09 (0.04; 0.14)	0.001	0.04 (−0.01; 0.09)	0.14	−0.02 (−0.07; 0.04)	0.56
Model 3	0.08 (0.02; 0.13)	0.004	0.03 (−0.02; 0.08)	0.27	−0.02 (−0.07; 0.04)	0.54
Model 4	0.08 (0.03; 0.13)	0.003	0.02 (−0.04; 0.08)	0.47	0.01 (−0.04; 0.07)	0.69
**Fatty liver index < 60** (*n* = 3324)						
Model 1	0.01 (−0.03; 0.04)	0.61	0.03 (−0.01; 0.06)	0.11	−0.02 (−0.06; 0.01)	0.21
Model 2	0.03 (−0.01; 0.06)	0.13	0.03 (−0.01; 0.07)	0.08	0.01 (−0.03; 0.04)	0.63
Model 3	0.02 (−0.01; 0.06)	0.21	0.03 (−0.01; 0.07)	0.08	0.01 (−0.02; 0.04)	0.57
Model 4	0.02 (−0.02; 0.05)	0.35	0.03 (−0.01; 0.07)	0.12	0.01 (−0.03; 0.04)	0.86

Model 1: Crude; Model 2: Adjusted for age and sex; Model 3: As model 2, additionally adjusted for waist circumference, eGFR, and urinary albumin excretion; Model 4: As model 3, additionally adjusted for alcohol intake, smoking, diabetes, and positive anti-TPO antibodies.

## Data Availability

The raw data supporting the conclusions of this article will be made available by the authors on request.

## Supplementary Materials

The following supporting information can be downloaded at: https://www.mdpi.com/article/10.3390/ijms262110525/s1.

## Abbreviations

ANOVA, analysis of variance; BMI, body mass index; CI, confidence intervals; CV, coefficient of variation; CVD, cardiovascular disease; EDTA, ethylenediaminetetraacetic acid; FLI, fatty liver index; FT4, free thyroxine; FT, free triiodothyronine; HDL-C, high density lipoprotein cholesterol; LDL, low density lipoproteins; MASLD, metabolic dysfunction-associated steatotic liver disease; NMR, nuclear magnetic resonance spectroscopy; PREVEND, Prevention of Renal and Vascular End Stage Disease; STROBE, Strengthening the Reporting of Observational Studies in Epidemiology; TMA, trimethylamine; TMAO, trimethylamine-N-oxide; TSH, thyroid stimulating hormone.
