# Supplementary material for: Lower Thyroid Function and Higher Plasma Choline: Effect Modification by Metabolic Dysfunction-Associated Steatotic Liver Disease
Source: Int J Mol Sci. 2025 Oct 29;26(21):10525. doi: 10.3390/ijms262110525 (PMC12608030; doi:10.3390/ijms262110525)
Supplement: Supplementary file 1 [file ijms-26-10525-s001.zip › ijms-3913811-supplementary.pdf]

Supplementary Materials

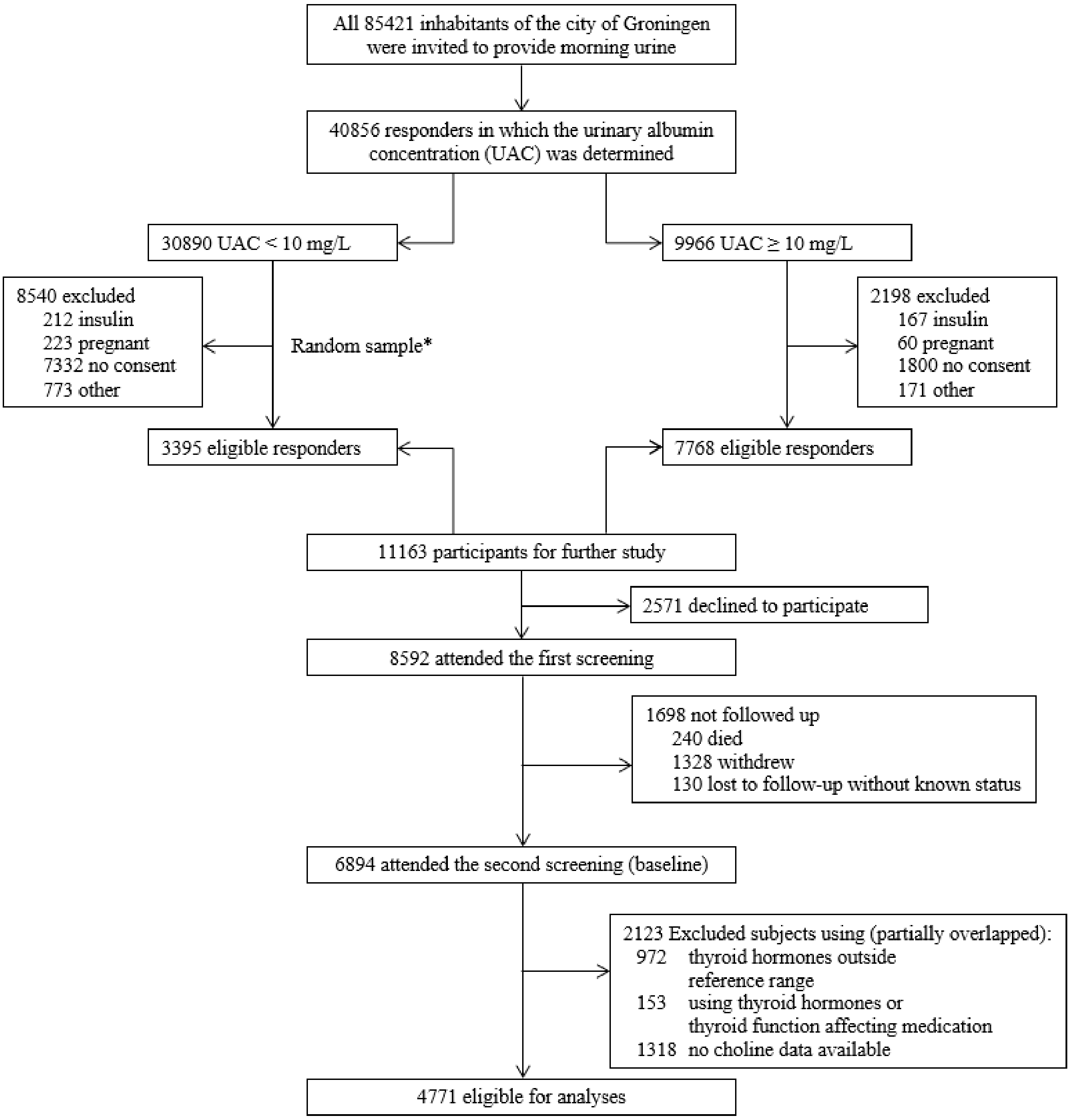

Figure S1. Study flowchart. *In the original design of the PREVEND study, the size of the random sample was arbitrarily set at 3395 (out of the 22,350 eligible participants) to obtain a total cohort size of ~10,000, taking into account a 15% nonparticipation rate.

**Table S1.** Linear regression analyses of thyroid stimulating hormone (TSH) with choline, TMAO, and betaine according to sex.						
	**Choline**		**TMAO**		**Betaine**	
**Males (n=2427)**						
**Model**	Std. β (95% CI)	*P-value*	*Std. β (95% CI)*	*P-value*	*Std. β (95% CI)*	*P-value*
**Model 1**	0.05 (0.01; 0.09)	0.017	0.03 (-0.01; 0.07)	0.16	-0.01 (-0.04; 0.04)	0.86
**Model 2**	0.05 (0.01; 0.09)	0.008	0.03 (-0.01; 0.07)	0.11	-0.01 (-0.04; 0.04)	0.90
**Model 3**	0.04 (0.01; 0.09)	0.032	0.03 (-0.01; 0.08)	0.13	0.01 (-0.04; 0.05)	0.77
**Model 4**	0.05 (0.01; 0.09)	0.026	0.03 (-0.01; 0.08)	0.13	0.01 (-0.03; 0.04)	0.67
**Females (n=2344)**						
**Model 1**	0.04 (-0.01; 0.08)	0.061	0.04 (-0.01; 0.08)	0.099	-0.01 (-0.05; 0.03)	0.72
**Model 2**	0.04 (0.01; 0.08)	0.049	0.04 (-0.01; 0.08)	0.081	-0.01 (-0.05; 0.03)	0.73
**Model 3**	0.03 (-0.01; 0.07)	0.15	0.02 (-0.03; 0.06)	0.47	0.01 (-0.04; 0.05)	0.85
**Model 4**	0.03 (-0.01; 0.08)	0.12	0.02 (-0.02; 0.06)	0.39	0.01 (-0.04; 0.05)	0.91

Model 1: Crude.

Model 2: Adjusted for age and sex.

Model 3: As model 2, additionally adjusted for waist circumference, eGFR and urinary albumin excretion, alcohol intake, and smoking.

Model 4: As model 3, additionally adjusted for positive anti-TPO antibodies.

**Table S2**. Linear regression analyses of thyroid stimulating hormone (TSH) with choline, TMAO, and betaine according to anti-TPO positivity.						
	**Choline**		**TMAO**		**Betaine**	
**Anti-TPO positive (n=367)**						
**Model**	Std. β (95% CI)	P-value	Std. β (95% CI)	P-value	Std. β (95% CI)	P-value
**Model 1**	0.04 (-0.07; 0.14)	0.48	0.09 (-0.02; 0.20	0.093	-0.04 (-0.15; 0.06)	0.42
**Model 2**	0.05 (-0.05; 0.15)	0.30	0.09 (-0.02; 0.20	0.083	-0.02 (-0.12; 0.09)	0.72
**Model 3**	0.05 (-0.05; 0.16)	0.33	0.06 (-0.05; 0.17)	0.27	-0.01 (-0.11; 0.10)	0.77
**Model 4**	0.05 (-0.05; 0.16)	0.33	0.06 (-0.05; 0.17)	0.27	-0.01 (-0.11; 0.10)	0.66
**Anti-TPO negative (n=4400)**						
**Model 1**	0.03 (0.01; 0.06)	0.034	0.03 (-0.01; 0.06)	0.070	-0.02 (-0.05; 0.01)	0.19
**Model 2**	0.05 (0.02; 0.07)	0.001	0.03 (0.01; 0.06)	0.042	-0.01 (-0.03; 0.03)	0.81
**Model 3**	0.04 (0.01; 0.06)	0.020	0.02 (-0.01; 0.05)	0.17	0.01 (-0.04; 0.05)	0.82
**Model 4**	0.04 (0.01; 0.06)	0.020	0.02 (-0.01; 0.05)	0.17	0.01 (-0.04; 0.05)	0.81

Model 1: Crude.
Model 2: Adjusted for age and sex.
Model 3: As model 2, additionally adjusted for waist circumference, eGFR and urinary albumin excretion, alcohol intake, and smoking.
Model 4: As model 3, additionally adjusted for positive anti-TPO antibodies.

Table S3. Linear regression analyses of thyroid stimulating hormone (TSH) with choline, TMAO, and betaine after excluding highest 5% percentile of choline values.

	**Choline**		**TMAO**		**Betaine**	
**Model**	**Std. β (95% CI)**	**P value**	**Std. β (95% CI)**	**P value**	**Std. β (95% CI)**	**P value**
**Model 1**	0.03 (-0.01; 0.05)	0.097	0.03 (-0.01; 0.06)	0.077	-0.03 (-0.06; 0.01)	0.070
**Model 2**	0.04 (0.01; 0.07)	0.004	0.03 (0.01; 0.06)	0.049	-0.01(-0.03; 0.03)	0.83
**Model 3**	0.03 (-0.01; 0.06)	0.054	0.02 (-0.01; 0.05)	0.19	0.01 (-0.03; 0.03)	0.86
**Model 4**	0.03 (0.01; 0.06)	0.036	0.02 (-0.01; 0.05)	0.15	0.01 (-0.03; 0.03)	0.80

Model 1: Crude.
Model 2: Adjusted for age and sex.
Model 3: As model 2, additionally adjusted for waist circumference, eGFR and urinary albumin excretion, alcohol intake, and smoking.
Model 4: As model 3, additionally adjusted for positive anti-TPO antibodies.

Table S4. Sensitivity analyses of the linear regression analyses of thyroid stimulating hormone (TSH) with choline, TMAO, and betaine, with additional adjustment for C-reactive protein.

	**Choline**		**TMAO**		**Betaine**	
**Model**	**Std. β (95% CI)**	**P-value**	**Std. β (95% CI)**	**P-value**	**Std. β (95% CI)**	**P-value**
**Base**	0.04 (0.01; 0.07)	0.012	0.03 (-0.01; 0.06)	0.094	0.02 (-0.03; 0.03)	0.82
**Base + CRP**	0.03 (0.01; 0.07)	0.031	0.02 (-0.01; 0.06)	0.11	0.01 (-0.02; 0.04)	0.55

Base is adjusted for age, sex, waist circumference, eGFR and urinary albumin excretion, alcohol intake, smoking, and positive anti-TPO antibodies.
